# Ultraprocessed Foods Associated With Depressive Symptoms Among Chinese Adolescents: Cross-Sectional Study

**DOI:** 10.2196/75061

**Published:** 2025-10-20

**Authors:** Honglv Xu, Jieru Yang, Dongyue Hu, Xiaoxiao Li, Feihui Zhou

**Affiliations:** 1 School of Medicine Kunming University kunming China

**Keywords:** adolescents, depressive symptoms, eating behavior, mental health, ultraprocessed food

## Abstract

**Background:**

Ultraprocessed food (UPF) consumption is very common among adolescents. Previous studies have suggested an association between UPF consumption and adolescent mental health problems, but studies on multiethnic adolescents in China are rare.

**Objective:**

This study investigated the association between UPF consumption and depressive symptoms in multiethnic adolescents in Yunnan Province, China.

**Methods:**

A cluster sampling of 8500 middle school students from 11 counties in Yunnan Province was conducted. The Chinese version of the Depression Anxiety Stress Scale-21 was administered to assess depressive symptoms, and a food frequency questionnaire was used to collect UPF consumption data. A generalized linear model was used to analyze the association between UPF consumption and depressive symptoms, and a restricted cubic spline was used to fit the correlation plot. The association analysis was performed after adjusting for demographic and confounding variables that might have influenced depressive symptoms.

**Results:**

The detection rates of depressive symptoms in adolescents were 28.3% (2402/8500) for the sample, 25.5% (1069/4184) for males, and 30.9% (1333/4316) for females. After adjusting for confounding variables, instant food (all participants: β=.22, 95% CI 0.13-0.32; *P*<.001; males: β=.15, 95% CI 0.03-0.27; *P*=.01; females: β=.29, 95% CI 0.15-0.43; *P*<.001), carbonated beverage (β=.20, 95% CI 0.12-0.29; *P*<.001; β=.15, 95% CI 0.04-0.26; *P*=.006; β=.29, 95% CI 0.16-0.43; *P*<.001;), energy beverage (β=.21, 95% CI 0.10-0.32; *P*<.001; β=.22, 95% CI 0.08-0.36; *P*=.002; β=.22, 95% CI 0.04-0.40; *P*=.01), and other beverage (β=.24, 95% CI 0.14-0.34; *P*<.001; β=.21, 95% CI 0.08-0.35; *P*=.002; β=.25, 95% CI 0.10-0.41; *P*=.001) consumption were associated with depressive symptoms for all participants, males, and females, respectively. Females were more sensitive to the association between UPF consumption and depressive symptoms. Moreover, female interaction with instant foods (β=.31, 95% CI 0.18-0.44; *P*<.001), carbonated beverages (β=.27, 95% CI 0.15-0.39; *P*<.001), energy beverages (β=.18, 95% CI 0.01-0.35; *P*=.04), and other sugar-sweetened beverages (β=.17, 95% CI 0.02-0.32; *P*=.03) was associated with depressive symptoms, respectively.

**Conclusions:**

These results indicated that UPF consumption was related to depressive symptoms in Yunnan multiethnic adolescents. Health education and behavioral interventions that reduce UPF consumption could protect adolescents from depressive symptoms.

## Introduction

Depression is one of the most common mental illnesses in the world. In 2008, the World Health Organization ranked major depression as the third leading cause of disease burden worldwide and projected that the disease would rank first by 2030 [1]. The Global Burden of Disease Study from 1990 to 2019 shows that depression rates are on the rise [2]. Recently, mental health problems among adolescents have become a widespread concern [3] and are a main disease burden for adolescents [4]. Adolescence is a peak period for mental health problems [5]. Depression is the primary cause of disability in adolescents [6]. Depressive symptoms are the most common psychopathological symptoms in adolescents and significantly increase self-injury risk (odds ratio [OR] 2.19, 95% 2.03-2.36) and death by suicide (OR 2.67, 95% CI 1.72-4.14) [3]. A systematic review and meta-analysis demonstrated that the point prevalence of elevated depressive symptoms among adolescents increased from 24% (95% CI 19%-28%) between 2001 and 2010 to 37% (95% CI 32%-42%) between 2011 and 2020 [7]. A German study reported that the prevalence of depressive symptoms in adolescents aged 12-17 years was 8.2% (95% CI 6.5%-9.9%) [8]. In a UK school-based survey, the prevalence of depressive symptoms among 11- to 16-year-olds ranged from 24% to 32% [9]. The prevalence rates of depressive symptoms among adolescents may be higher in developing countries than in developed countries; the rates were 52.3% in India [10] and 26.2% among Malaysian secondary school students [11]. A meta-analysis of Chinese children and adolescents showed a total prevalence of depressive symptoms of 19.85% (95% CI 14.75%-24.96%), and the prevalence rates in the eastern, central, western, and northeastern regions were 17.8%, 23.7%, 22.7%, and 14.5%, respectively [12]. Our previous sample survey of 10- to 20-year-olds from 4 cities in China determined a 27.3% detection rate for depressive symptoms [13]. Current studies claim depressive symptoms are affected by various factors such as academic pressure, growth and development, eating behavior, physical activity, and social media use [3,14,15].

The global consumption of ultraprocessed foods (UPF) has rapidly increased. In 2010, the NOVA food classification system was proposed [16]. After undergoing physical, biological, and chemical processes and containing multiple ingredients and additives, UPF is separated from nature before consumption or preparation as dishes [16]. UPF has the advantages of attractive packaging, ease of consumption, attractive taste, and high palatability, and has gained popularity, especially among adolescents [17]. UPF consumption by adolescents in developed countries is higher; the proportion of energy intake from UPF in American adolescents is higher than that in adults [18]. The UPF energy intake in British middle school students accounts for 77.8% of their total lunch energy intake [19]. UPF consumption among adolescents in developing countries has also attracted widespread attention. According to a Romanian survey, 18- to 23-year-olds generally consume pastries (41.4%), while sweets (12.4%), pastries (11.1%), and sugar-sweetened beverages (SSB; 11.2%) are highly addictive, and males consume more than females [20]. Data from the Global School Health Survey of 9 Southeast Asian countries suggest that 40% of 12- to 15-year-olds consume carbonated beverages [21]. Adolescents aged 14-17 years in Spain consume 7.72 UPF servings per day; the reported consumption rates for processed meats, chocolate products, snacks, chocolate drinks, and soft drinks were 52.14%, 49.29%, 48.21%, 46.07%, and 33.57%, respectively [22]. UPF consumption by adolescents is associated with social isolation, diet globalization, and parental rearing style [23].

Existing research demonstrates that unhealthy eating behaviors and eating patterns of adolescents are potentially associated with mental health issues [24]. UPF consumption is associated with depressive symptoms (OR 1.44, 95% CI 1.14-1.82) [25] and significantly associated with an increased risk of depression (relative risk 1.31, 95% CI 1.21-1.41) [26]. Western dietary patterns (sweetened beverages, processed or junk foods, and foods rich in saturated fatty acids) have been linked to an increased risk of depression among adolescents [27]. SSB, fried foods, processed meats, and baked products are associated with an increased risk of depression. SSB, sweets, and snack consumption are associated with depressive symptoms in adolescents (OR 1.62, 95% CI 1.35-1.95) [28]. Studies in China, the United Kingdom, Brazil, and other countries have found that the consumption of sweets, biscuits, snacks, and fast foods and depressive symptoms in adolescents are significantly positively correlated [29-32]. For instance, in the Brazilian National School Health Survey, boys and girls who consumed ≥6 UPF reported more depressive symptoms and other psychopathologies than those who consumed ≤3 UPF 24 hours before the survey (boys: β=.27, girls: β=.31) [33].

Although several studies have explored the association between UPF consumption and depressive symptoms in adolescents, their findings have been inconsistent. Studies have shown that the consumption of soft drinks and processed meat products is not associated with depressive symptoms in adolescents [30]. There are limited studies on the association between UPF consumption and depressive symptoms of adolescents in China, and no studies on multiethnic adolescents in China have been conducted. Chinese multiethnic adolescents mainly live in the border areas of China and are influenced by the dietary culture of neighboring countries and multiethnic groups; thus, their UPF consumption may be higher. Therefore, this study investigated the association between UPF consumption and depressive symptoms in Chinese multiethnic adolescents.

## Methods

### Study Design

This is a cross-sectional study. This study is part of a behavior and subhealth study of Yunnan adolescents that aims to explore the association between lifestyle factors and physical and mental subhealth among adolescents in Yunnan, China, through a series of cohort studies [34].

### Study Setting and Sample

**Figure 1:**
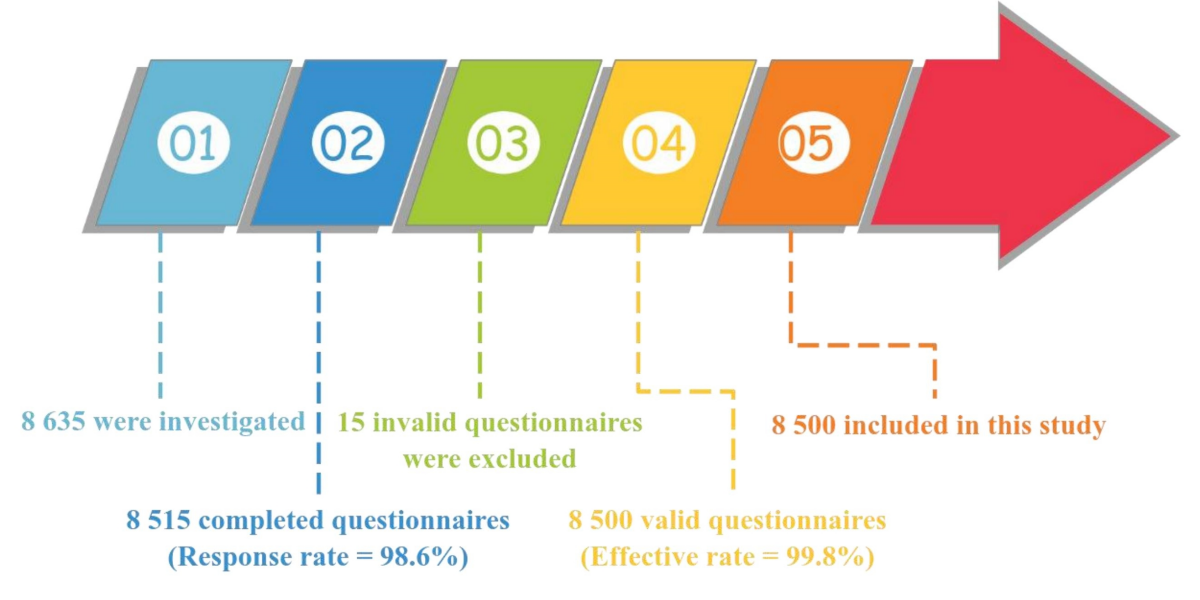
Flow chart of participants.

From October to December 2022, a sample questionnaire survey was conducted with first-year (grade 7) middle school students from 11 counties in Yunnan Province, China. Yunnan Province lies along China’s southwestern border with Myanmar, Laos, and Vietnam. Yunnan has 26 ethnic groups and 25 ethnic minority groups. A total of 8635 multiethnic adolescents were surveyed, and 8515 questionnaires were completed, for a 98.6% response rate. There were 8500 valid questionnaires; the effective response rate was 99.8%. The average age of participants was 12.64 (SD 0.63; range 12-15) years; 4184 (49.2%) were males and 4136 (48.8%) were females; and 6358 (74.8%) were from rural areas and 2142 (25.2%) were from urban areas. There were 3856 (45.3%) Han and 4659 (54.7%) ethnic minority participants. Figure 1 presents the flowchart of participants. Tables 1 and 2 present the distributions of the demographic variables.

**Table 1:** Distribution of depressive symptoms in 12-year-old adolescents.

Variables			Normal, n (%)		Depressive symptoms, n (%)		Total, n (%)		Chi-square (*df*)		*P* value			
**Sex**										18.3 (1)		<.001		
	Male	1280 (47.2)		388 (39.3)		1668 (45.1)								
	Female	1431 (52.8)		599 (60.7)		2030 (54.9)								
**Ethnicity**										14.2 (4)		.007		
	Han	1143 (42.2)		372 (37.7)		1515 (41)								
	Hani	680 (25.1)		255 (25.9)		935 (25.3)								
	Yi	460 (17)		168 (17)		628 (17)								
	Lisu	76 (2.8)		48 (4.9)		124 (3.4)								
	Other	352 (13)		144 (14.6)		496 (13.4)								
**The number of close friends**										110.2 (2)		<.001		
	0-2	230 (8.5)		192 (19.5)		422 (11.4)								
	3-4	418 (15.4)		198 (20.1)		616 (16.7)								
	≥5	2063 (76.1)		597 (60.5)		2660 (71.9)								
**Residence**										4.6 (1)		.03		
	Rural	1822 (67.2)		700 (70.9)		2522 (68.2)								
	Urban	889 (32.8)		287 (29.1)		1176 (31.8)								
**The only child in the family**										5.8 (1)		.02		
	Yes	406 (15)		180 (18.2)		586 (15.8)								
	No	2305 (85)		807 (81.8)		3112 (84.2)								
**Family type**										16.6 (3)		.001		
	2-parent family	2259 (83.3)		765 (77.5)		3024 (81.8)								
	1-parent family	250 (9.2)		121 (12.3)		371 (10)								
	Combined family	171 (6.3)		84 (8.5)		255 (6.9)								
	Other	31 (1.1)		17 (1.7)		48 (1.3)								
**Father’s education level**										9.3 (4)		.05		
	Illiteracy	268 (9.9)		126 (12.8)		394 (10.7)								
	Elementary school	567 (20.9)		197 (20)		764 (20.7)								
	Secondary school	1295 (47.8)		470 (47.6)		1765 (47.7)								
	High school	372 (13.7)		136 (13.8)		508 (13.7)								
	University	209 (7.7)		58 (5.9)		267 (7.2)								
**Mother’s education level**										7.4 (4)		.12		
	Illiteracy	457 (16.9)		197 (20)		654 (17.7)								
	Elementary school	588 (21.7)		221 (22.4)		809 (21.9)								
	Secondary school	1148 (42.3)		398 (40.3)		1546 (41.8)								
	High school	328 (12.1)		117 (11.9)		445 (12)								
	University	190 (7)		54 (5.5)		244 (6.6)								
**Father’s occupation**										12.7 (5)		.03		
	Civil servant	1186 (43.7)		459 (46.5)		1645 (44.5)								
	Worker	215 (7.9)		63 (6.4)		278 (7.5)								
	Staff	569 (21)		211 (21.4)		780 (21.1)								
	Merchant	260 (9.6)		64 (6.5)		324 (8.8)								
	Farmer	109 (4)		40 (4.1)		149 (4)								
	Other	372 (13.7)		150 (15.2)		522 (14.1)								
**Mother’s occupation**										8.8 (5)		.12		
	Civil servant	1285 (47.4)		503 (51)		1788 (48.4)								
	Worker	178 (6.6)		48 (4.9)		226 (6.1)								
	Staff	345 (12.7)		127 (12.9)		472 (12.8)								
	Merchant	298 (11)		88 (8.9)		386 (10.4)								
	Farmer	140 (5.2)		46 (4.7)		186 (5)								
	Other	465 (17.2)		175 (17.7)		640 (17.3)								

**Table 2:** Distribution of depressive symptoms in 13- to 15-year-old adolescents.

Variables		Normal, n (%)	Depressive symptoms, n (%)	Total, n (%)	Chi-square (*df*)		*P* value	
**Sex**						14.7 (1)		<.001
	Male	1835 (54.2)	681 (48.1)	2516 (52.4)				
	Female	1552 (45.8)	734 (51.9)	2286 (47.6)				
**Ethnicity**						98.1 (4)		<.001
	Han	1766 (52.1)	575 (40.6)	2341 (48.8)				
	Hani	440 (13)	210 (14.8)	650 (13.5)				
	Yi	550 (16.2)	201 (14.2)	751 (15.6)				
	Lisu	269 (7.9)	208 (14.7)	477 (10)				
	Other	362 (10.7)	221 (15.6)	583 (12.1)				
**The number of close friends**						90.3 (2)		<.001
	0-2	278 (8.2)	245 (17.3)	523 (10.9)				
	3-4	516 (15.2)	229 (16.2)	745 (15.5)				
	≥5	2593 (76.6)	941 (66.5)	3534 (73.6)				
**Residence**						0.3 (1)		.62
	Rural	2712 (80)	1124 (79.4)	3836 (79.9)				
	Urban	675 (19.9)	291 (20.6)	966 (20.1)				
**The only child in the family**						2.6 (1)		.11
	Yes	455 (13.4)	215 (15.2)	670 (14)				
	No	2932 (86.6)	1200 (84.8)	4132 (86)				
**Family type**						37.9 (3)		<.001
	2-parent family	2794 (82.5)	1060 (74.9)	3854 (80.3)				
	1-parent family	312 (9.2)	179 (12.7)	491 (10.2)				
	Combined family	227 (6.7)	135 (9.5)	362 (7.5)				
	Other	54 (1.6)	41 (2.9)	95 (2)				
**Father’s education level**						15.7 (4)		.004
	Illiteracy	448 (13.2)	242 (17.1)	690 (14.4)				
	Elementary school	831 (24.5)	361 (25.5)	1192 (24.8)				
	Secondary school	1569 (46.3)	597 (42.2)	2166 (45.1)				
	High school	387 (11.4)	161 (11.4)	548 (11.4)				
	University	152 (4.5)	54 (3.8)	206 (4.3)				
**Mother’s education level**						24.9 (4)		<.001
	Illiteracy	684 (20.2)	362 (25.6)	1046 (21.8)				
	Elementary school	869 (25.7)	367 (25.9)	1236 (25.7)				
	Secondary school	1422 (42)	504 (35.6)	1926 (40.1)				
	High school	291 (8.6)	136 (9.6)	427 (8.9)				
	University	121 (3.6)	46 (3.3)	167 (3.5)				
**Father’s occupation**						8.4 (5)		.14
	Civil servant	1813 (53.5)	715 (50.5)	2528 (52.6)				
	Worker	159 (4.7)	65 (4.6)	224 (4.7)				
	Staff	709 (20.9)	309 (21.8)	1018 (21.2)				
	Merchant	243 (7.2)	92 (6.5)	335 (7)				
	Farmer	80 (2.4)	40 (2.8)	120 (2.5)				
	Other	383 (11.3)	194 (13.7)	577 (12)				
**Mother’s occupation**						11.3 (5)		.045
	Civil servant	2047 (60.4)	825 (58.3)	2872 (59.8)				
	Worker	137 (4)	46 (3.3)	183 (3.8)				
	Staff	403 (11.9)	172 (12.2)	575 (12)				
	Merchant	268 (7.9)	104 (7.3)	372 (7.7)				
	Farmer	99 (2.9)	39 (2.8)	138 (2.9)				
	Other	433 (12.8)	229 (16.2)	662 (13.8)				

Cluster random sampling was used to conduct a questionnaire survey among adolescents in 11 counties of Yunnan Province, and 1-2 middle schools were selected from each county. SPSS software (version 23.0; IBM Corp) was used for random sampling. All students in grade 7 from the sampled middle schools were included in the survey.

The survey included demographic variables, eating behaviors, anxiety symptoms, and confounding factors that may influence depressive symptoms in adolescents, such as family changes, hospital experiences, screen time, smoking, drinking behavior, physical activity, family history of depression, learning stress, and the impact of COVID-19. The selection of these confounding factors was based on references and previous research findings of the research group.

A pilot test was conducted on 131 middle school students before the field survey to assess participant understanding of the questionnaire. The pilot test results showed that middle school students were able to fully understand the content of the questionnaire, and the average time taken to complete the questionnaire was 16 minutes. The adolescents gathered in a classroom to complete the survey. Trained investigators were on-site to answer questions from adolescents face-to-face. Adolescents completed the paper version of the questionnaire independently, which takes approximately 15-20 minutes.

### Measures

#### Covariates

The demographic variables included sex, age, ethnicity, only child status, family residence, self-rated family economic status, family type, number of close friends, parental education, and parental occupation.

#### Depressive Symptom Assessment

The Children’s Depression Inventory (CDI) was used to assess depressive symptoms in adolescents [35]. The CDI is applicable for the self-assessment of depressive symptoms in children and adolescents between the ages of 7 and 17 years; it is based on self-perception within the past 2 weeks. The CDI consists of 27 items, each of which is scored on a 3-point scale of “occasionally,” “often,” and “always” (0-2 points), respectively. The total CDI score ranges from 0 to 54; higher scores indicate more severe depressive symptoms. In this study, a total score of ≥19 was considered positive for depressive symptoms, and a score <19 was considered negative for depressive symptoms [35]. Research on Chinese children and adolescents aged 7-17 years shows that the CDI has good reliability and validity with a Cronbach α of 0.84 [36]. In this study, Cronbach α for the CDI was 0.853.

#### UPF Consumption Assessment

The eating frequency questionnaire prepared by our research group, which has good reliability and validity, was used to evaluate the eating behavior of adolescents [37]. According to the NOVA food classification system proposed by Monteiro [16], UPF refers to food that is separated from nature before being consumed or prepared as dishes, undergoes physical, biological, or chemical processes, and contains multiple ingredients and additives, for example, soft drinks, ice cream, packaged sweets, salty snacks, processed meat, and prepared frozen foods. This survey focused on the average weekly UPF consumption of adolescents in the previous month, including staple processed food (eg, fried bread stick, fried dough cake, 200 g per serving), processed meat (eg, ham, sausage, ham sausage, Chinese bacon, canned meat, beef jerky, chicken steak, 100 g per serving), processed eggs (eg, salted duck egg, spiced corned egg, 60 g per serving), preserved fruits (eg, preserved apple, 100 g per serving), instant foods (eg, instant noodles, 100 g per serving), salted snacks (refers to industrial production, packaging, and sales, as well as long-term preserved salty food; eg, spicy strip, salty crackers, 100 g per serving), sweet snacks (refers to industrial production, packaging, and sales, as well as long-term preserved sweet food; eg, chocolate, candy, 50 g per serving), SSB (eg, carbonated beverages, juice beverages, energy beverages, milk beverages [refers to a category of SSB made by blending milk with various additives, such as sugar, sweeteners, and acidifiers, eg, wang zai milk], 500 mL per bottle), other SSB (refers to SSB other than the sweet snacks, carbonated beverages, juice beverages, energy beverages, and milk beverages; eg, vitamin beverages, sports beverages, 500 mL per bottle). We used concise questions to assess UPF consumption frequency. For instance, how many bottles of Coca-Cola (500 mL per serving) did you drink per week in the last 1 month? There were 10 options: 0, 1, 2, 3, 4, 5, 6, 7, 8, and ≥9 times. Adolescents choose their consumption patterns based on their actual situations. The Cronbach α of the dietary frequency questionnaire in this study was 0.935.

### Data Analysis

We used EpiData (version 3.0; EpiData Association) to build the database, and SPSS software (version 23.0; IBM Inc) and R software (version 4.3.1; R Foundation for Statistical Computing) were used for statistical analysis. In the SPSS software, descriptive statistics, a chi-square test, and a generalized linear model (GLM) analysis were performed. Restricted cubic spline (RCS) analysis was performed using R software. A chi-square test was used to compare the difference in depressive symptoms detection rate among middle school students with different demographic characteristics. A GLM and RCS were used to analyze the association between UPF consumption and depressive symptoms. In the model, the UPF consumption frequency (independent variable) and depressive symptom score (dependent variable) were continuous variables. An association analysis was performed after adjusting for demographic and confounding variables that might have influenced depressive symptoms. The association between UPF consumption and depressive symptoms was analyzed using the RCS, and an RCS correlation plot was fitted. A GLM was used to analyze the association between the interaction term of sex, UPF consumption, and depressive symptoms. The test standard was α=.05.

### Ethical Considerations

This study was approved by the Ethics Committee of the School of Medicine, Kunming University (approval 20210222), and was carried out in accordance with the Declaration of Helsinki. Ethical guidelines were followed during all the stages of this study. The survey was conducted anonymously with informed consent from the schools, guardians, and students. All participants joined the study voluntarily, and no compensation was provided to them. All data were kept confidential, deidentified, and anonymous.

## Results

### Distribution of Depressive Symptoms in Adolescents With Different Demographic Variables

The total detection rate of depressive symptoms was 28.3% (2402/8500) among middle school students, 25.5% (1069/4184) among males, and 30.9% (1333/4316) among females; the difference was statistically significant (*χ*^2^_1_=29.83; *P*<.001). The detection rate of depressive symptoms in 12-year-olds was 26.7% (987/3698), and that in 13- to 15-year-olds was 29.5% (1415/4802); the difference was statistically significant (*χ*^2^_1_=7.95; *P*=.005). The stratified analysis by age (12 years and 13-15 years) showed statistically significant differences in the detection rate of depressive symptoms among 12-year-olds for all demographic variables except parental education level and maternal occupation. Among adolescents aged 13-15 years, there were statistically significant differences in the detection rates of depressive symptoms for other demographic variables except for only child, residence, and parental occupation (*P*<.05).

### Association of UPF Consumption With Depressive Symptoms in Adolescents

The consumption rates (frequency ≥1) of staple processed food, processed meat, processed eggs, preserved fruits, instant foods, salted snacks, sweet snacks, carbonated beverages, juice beverages, energy beverages, milk beverages and other SSB for adolescents are presented in Table 3.

**Table 3:** Consumption of ultraprocessed foods among adolescents.

Variable	Adolescents (N=8500), n (%)	Males (n=4184), n (%)	Females (n=4316), n (%)
Staple processed food	4712 (55.4)	2274 (54.3)	2438 (56.5)
Processed meat	5761 (67.8)	2870 (68.6)	2891 (67.0)
Processed eggs	3878 (45.6)	1943 (46.4)	1935 (44.8)
Preserved fruits	4387 (51.6)	2037 (48.7)	2350 (54.4)
Instant foods	5234 (61.6)	2648 (63.3)	2586 (59.9)
Salted snacks	5702 (67.1)	2759 (65.9)	2943 (68.2)
Sweet snacks	5271 (62.0)	2490 (59.5)	2781 (64.4)
Carbonated beverages	6022 (70.8)	3187 (76.2)	2835 (65.7)
Juice beverages	5612 (66.0)	2687 (64.2)	2925 (67.8)
Energy beverages	3716 (43.7)	2109 (50.4)	1607 (37.2)
Milk beverages	6025 (70.9)	2732 (65.3)	3293 (76.3)
Other SSB^a^	3739 (44.0)	1899 (45.4)	1840 (42.6)

^a^SSB: sugar-sweetened beverages.

Table 4 presents the results of the GLM analysis of the association between UPF consumption and depressive symptoms in adolescents after adjusting for variables (Model 2). Table S1 in Multimedia Appendix 1 shows the analysis results without adjusting for variables (Model 1). After adjusting for confounding variables, instant food (β=.22, 95% CI 0.13-0.32; *P*<.001), carbonated beverage (β=.20, 95% CI 0.12-0.29; *P*<.001), energy beverage (β=.21, 95% CI 0.10-0.32; *P*<.001), and other SSB (β=.24, 95% CI 0.14-0.34; *P*<.001) consumption were associated with depressive symptoms for all participants. The sex-stratified analysis showed that instant food (β=.15, 95% CI 0.03-0.27; *P*=.01), carbonated beverage (β=.15, 95% CI 0.04-0.26; *P*=.006), energy beverage (β=.22, 95% CI 0.08-0.36; *P*=.002), milk beverage (β=–0.14, 95% CI –0.26 to –0.02; *P*=.03), and other SSB (β=.21, 95% CI 0.08-0.35; *P*=.002) consumption was associated with depressive symptoms in males. In addition, instant food (β=.29, 95% CI 0.15-0.43; *P*<.001), carbonated beverage (β=.29, 95% CI 0.16-0.43; *P*<.001), energy beverage (β=.22, 95% CI 0.04-0.40; *P*=.01), and other SSB (β=.25, 95% CI 0.10-0.41; *P*=.001) consumption were associated with depressive symptoms in females.

**Table 4:** Association of ultraprocessed food consumption with depressive symptoms in adolescents. Adjusted for age, ethnicity, residence, sex, the only child in the family, parental occupation, family type, parental educational level, the number of close friends, self-perceived academic stress, self-perceived socioeconomic status, family changes, hospital experience, smoking, alcohol consumption, family history of depression, left-behind experience, physical activity, and the impact of COVID-19. The analysis of all participants also adjusted for sex.

Variable		β (95% CI)	SE	Wald chi-square (*df*)	*P* value	
**All** **participants**						
	Staple processed food	–.08 (–0.17 to 0.00)	0.04	3.7 (1)	.06	
	Processed meat	–.04 (–0.12 to 0.04)	0.04	1.0 (1)	.33	
	Processed eggs	–.04 (–0.14 to 0.05)	0.05	0.8 (1)	.38	
	Preserved fruits	–.04 (–0.14 to 0.05)	0.05	0.8 (1)	.37	
	Instant foods	.22 (0.13 to 0.32)	0.05	23.3 (1)	<.001	
	Salted snacks	–.03 (–0.10 to 0.05)	0.04	0.5 (1)	.50	
	Sweet snacks	–.04 (–0.12 to 0.04)	0.04	1.0 (1)	.31	
	Carbonated beverages	.20 (0.12 to 0.29)	0.04	23.0 (1)	<.001	
	Juice beverages	–.06 (–0.15 to 0.03)	0.05	1.6 (1)	.21	
	Energy beverages	.21 (0.10 to 0.32)	0.06	14.7 (1)	<.001	
	Milk beverages	–.07 (–0.15 to 0.02)	0.04	2.3 (1)	.13	
	Other SSB^a^	.24 (0.14 to 0.34)	0.05	20.8 (1)	<.001	
**Males**						
	Staple processed food	–.11 (–0.23 to 0.00)	0.06	3.8 (1)	.05	
	Processed meat	–.03 (–0.13 to 0.08)	0.05	0.3 (1)	.59	
	Processed eggs	.01 (–0.11 to 0.14)	0.06	0.0 (1)	.84	
	Preserved fruits	–.03 (–0.17 to 0.10)	0.07	0.2 (1)	.62	
	Instant foods	.15 (0.03 to 0.27)	0.06	6.1 (1)	.01	
	Salted snacks	.01 (–0.10 to 0.12)	0.05	0.0 (1)	.85	
	Sweet snacks	.00 (–0.12 to 0.11)	0.06	0.0 (1)	.94	
	Carbonated beverages	.15 (0.04 to 0.26)	0.05	7.6 (1)	.006	
	Juice beverages	–.02 (–0.14 to 0.10)	0.06	0.1 (1)	.76	
	Energy beverages	.22 (0.08 to 0.36)	0.07	9.9 (1)	.002	
	Milk beverages	–.14 (–0.26 to –0.02)	0.06	4.9 (1)	.03	
	Other SSB	.21 (0.08 to 0.35)	0.07	9.8 (1)	.002	
**Females**						
	Staple processed food	–.04 (–0.17 to 0.09)	0.07	0.4 (1)	.54	
	Processed meat	–.04 (–0.17 to 0.09)	0.06	0.4 (1)	.55	
	Processed eggs	–.09 (–0.24 to 0.05)	0.07	1.6 (1)	.20	
	Preserved fruits	–.06 (–0.21 to 0.08)	0.07	0.7 (1)	.40	
	Instant foods	.29 (0.15 to 0.43)	0.07	16.6 (1)	<.001	
	Salted snacks	–.07 (–0.19 to 0.04)	0.06	1.6 (1)	.20	
	Sweet snacks	–.09 (–0.20 to 0.02)	0.06	2.6 (1)	.11	
	Carbonated beverages	.29 (0.16 to 0.43)	0.07	18.9 (1)	<.001	
	Juice beverages	–.12 (–0.26 to 0.02)	0.07	2.8 (1)	.10	
	Energy beverages	.22 (0.04 to 0.40)	0.09	6.0 (1)	.01	
	Milk beverages	.00 (–0.13 to 0.12)	0.06	0.0 (1)	.96	
	Other SSB	.25 (0.10 to 0.41)	0.08	10.1 (1)	.001	

^a^SSB: sugar-sweetened beverages.

Tables 5 and 6 present the association between the interaction of sex, UPF consumption, and depressive symptoms in adolescents. The results showed that female×instant foods (β=.31, 95% CI 0.18-0.44; *P*<.001), female×carbonated beverages (β=.27, 95% CI 0.15-0.39; *P*<.001), female×energy beverages (β=.18, 95% CI 0.01-0.35; *P*=.04), and female×other SSB (β=.17, 95% CI 0.02-0.32; *P*=.03) were associated with depressive symptoms, respectively. However, no association was observed between the interaction of these UPFs and male and depressive symptoms.

**Table 5:** Association between interaction of sex, ultraprocessed food consumption, and depressive symptoms in adolescents (Model 1: unadjusted for variables).

Variable	β (95% CI)	SE	Wald chi-square (*df*)	*P* value
Male×instant foods	.14 (0.01 to 0.26)	0.06	4.7 (1)	.03
Female×instant foods	.44 (0.30 to 0.58)	0.07	38.6 (1)	<.001
Male×carbonated beverages	.09 (–0.02 to 0.19)	0.05	2.5 (1)	.12
Female×carbonated beverages	.31 (0.18 to 0.44)	0.07	21.7 (1)	<.001
Male×energy beverages	.12 (–0.03 to 0.27)	0.08	2.4 (1)	.12
Female×energy beverages	.19 (0.00 to 0.37)	0.09	3.8 (1)	.05
Male×other SSB^a^	.15 (0.01 to 0.30)	0.07	4.4 (1)	.04
Female×other SSB	.18 (0.02 to 0.34)	0.08	4.6 (1)	.03

^b^SSB: sugar-sweetened beverages.

**Table 6:** Association between interaction of sex, ultraprocessed food consumption, and depressive symptoms in adolescents (Model 2: adjusted for age, ethnicity, residence, the only child in the family, parental occupation, family type, parental educational level, the number of close friends, self-perceived academic stress, self-perceived socioeconomic status, family changes, hospital experience, smoking, alcohol consumption, family history of depression, left-behind experience, physical activity, and the impact of COVID-19).

Variable	β (95% CI)	SE	Wald chi-square (*df*)	*P* value
Male×instant foods	.11 (–0.01 to 0.22)	0.06	3.3 (1)	.07
Female×instant foods	.31 (0.18 to 0.44)	0.07	21.6 (1)	<.001
Male×carbonated beverages	.06 (–0.05 to 0.15)	0.05	1.2 (1)	.28
Female×carbonated beverages	.27 (0.15 to 0.39)	0.06	19.4 (1)	<.001
Male×energy beverages	.12 (–0.02 to 0.25)	0.07	2.7 (1)	.10
Female×energy beverages	.18 (0.01 to 0.35)	0.09	4.1 (1)	.04
Male×other SSB^a^	.12 (–0.01 to 0.25)	0.07	3.1 (1)	.08
Female×other SSB	.17 (0.02 to 0.32)	0.08	5.0 (1)	.03

^a^SSB: sugar-sweetened beverages.

**Figure 2:**
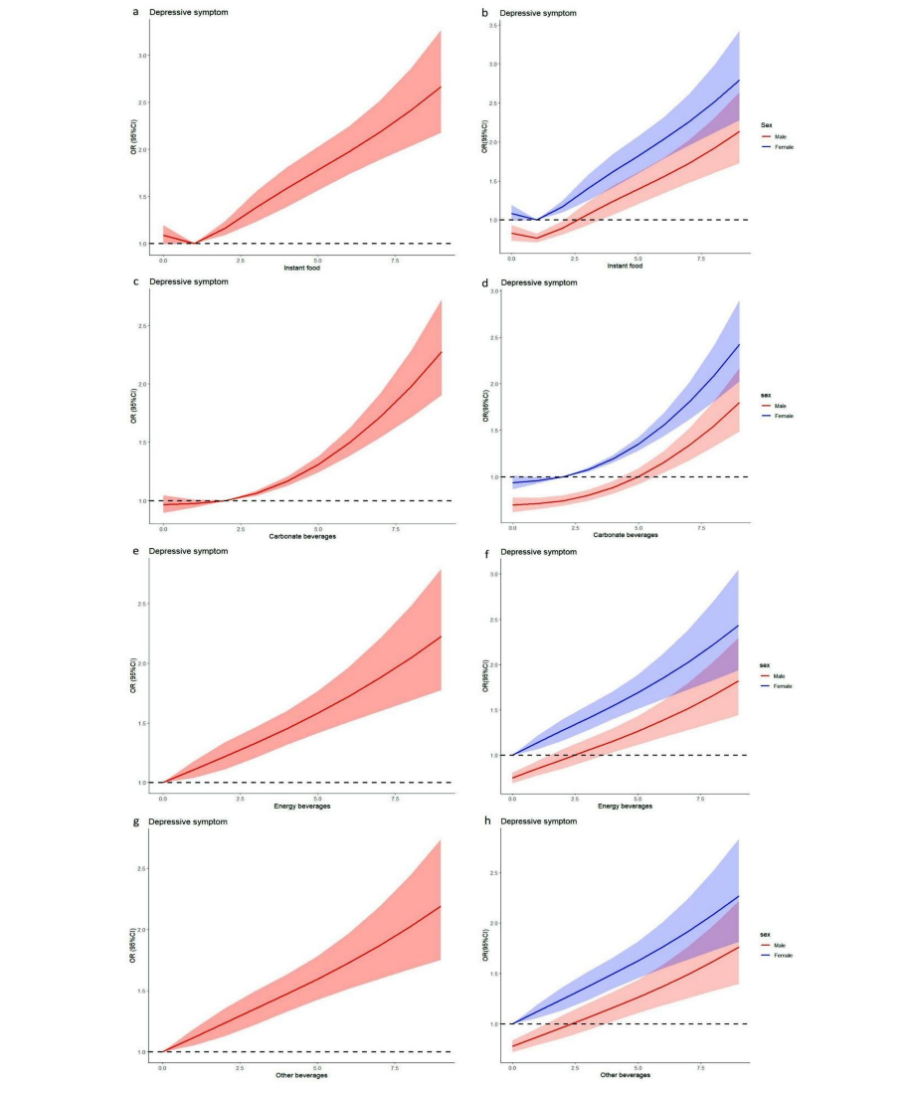
Association of ultraprocessed food consumption with depressive symptoms in adolescents. OR: odds ratio.

Figure 2 displays the results of the RCS analysis for the association between UPF consumption and depressive symptoms in adolescents. Overall, UPF consumption was significantly associated with depressive symptoms in adolescents, and females were more sensitive to UPF consumption (exposure) than males.

## Discussion

### Principal Findings

In this study, the detection rate for depressive symptoms among adolescents of multiple ethnic groups in Yunnan was higher (2402/8500, 28.3%), which is consistent with the results of our previous study on adolescents in 4 cities in China (27.3%) [13]. This detection rate was higher than that in a meta-analysis of middle school students in mainland China (24.3%) and that of middle school students in eastern China (23.3%) [38,39]; however, it was lower than that for Bangladeshi adolescents (36.6%) [40] and Indian adolescents (52.3%) [10]. Our data showed the detection rate of depressive symptoms for adolescents aged 13-15 years (1415/4802, 29.5%) was higher than that for adolescents aged 12 years (987/3698, 26.7%). A school-based survey in the United Kingdom determined that the detection rate of depressive symptoms among 15- to 16-year-olds (32%) was higher than that among 11- to 12-year-olds (24%) [9]. In addition, we found the detection rate of depressive symptoms in females (1333/4316, 30.9%) was significantly higher than that for males (1069/4184, 25.5%), which is consistent with the conclusions of other studies [40]. For example, a Bangladeshi study reported a significantly higher prevalence of depressive symptoms among female adolescents than male adolescents (32.5% vs 25.3%) [40]. The higher prevalence of depressive symptoms among female adolescents is consistent worldwide. Numerous studies have suggested that depressive symptoms increase during adolescence, adolescent females are more susceptible to depressive symptoms, and sex differences appear only during adolescence [41]. A meta-analysis found that sex differences peaked in adolescence (OR 3.02, 95% CI 2.76-3.30 for ages 13-15 years), then declined and remained stable in adulthood [42]. Moreover, the longitudinal study of Australian children also found that between the ages of 4 and 14 years, females were more likely than males to be on a trajectory for increased depressive symptoms [43]. Therefore, schools and families need to pay more attention to depressive symptoms in adolescents, especially female adolescents.

Our study suggests that fast foods, carbonated beverages, energy beverages, and other SSB (except those listed in this study) were positively associated with depressive symptoms in both males and females. Other studies have also determined that UPF consumption is associated with depressive symptoms [25,29,31]. Our survey of Chinese college freshmen demonstrated that carbonated beverage and milk tea consumption were associated with depressive symptoms in males, whereas carbonated beverages, tea beverages, and milk tea consumption were associated with depressive symptoms in females [44]. Higher UPF consumption in Spanish adolescents aged 14-17 years was associated with a higher detection rate of depressive symptoms, and the higher the intake, the more pronounced the depressive symptoms [22]. The consumption of sweets, biscuits, snacks, and fast food among adolescents in England, Wales, and Northern Ireland was significantly positively associated with depressive symptoms [30]. Instant noodles, instant rice, and other instant foods are very popular among Chinese adolescents because they are easy to carry and eat. Our survey indicated that the consumption rate of instant foods such as instant noodles was as high as 61.6% (5234/8500), which has not been previously reported in China. Our previous research found an association between carbonated beverage and processed snack consumption and anxiety symptoms in Yunnan college students [45]. This was the first study to report a correlation between instant food consumption and depressive symptoms among Chinese multiethnic adolescents.

Furthermore, the data from this study showed that milk beverage consumption was negatively associated with depressive symptoms in males. The milk beverages in this study were also SSB; thus, we will further observe and evaluate these associations in a cohort study. The RCS analysis revealed that instant food, carbonated beverages, energy beverages, and other SSB were associated with depressive symptoms in males and females, and the association between UPF consumption and depressive symptoms in females was more sensitive. We further analyzed the interaction between sex and UPF and its association with depressive symptoms. The results indicated that females who interact with instant foods, carbonated beverages, energy beverages, and other SSBs were associated with depressive symptoms, respectively. However, no association was observed between the interaction of males and these 4 types of UPFs and depressive symptoms. The research data further support the view that females who consumed UPF were more likely to experience depressive symptoms. Therefore, when schools develop intervention measures to reduce depression symptoms among adolescents through dietary behavior intervention, they need to take into account sex-specific factors. For female adolescents, it is even more necessary to strengthen health education and behavioral intervention.

Several mechanisms by which UPF consumption is associated with depressive symptoms are possible. First, UPF consumption is associated with sleep dissatisfaction in adolescents [28]. The low sleep quality is a predictor of depressive symptoms in adolescents. Previous studies have suggested that the fourth quartile of UPF (44.3%-81.8% of total calories; prevalence ratio=1.14) was associated with a higher prevalence of poor sleep quality in adolescents [46]. A systematic review and meta-analysis showed that the higher the intake of UPF, the greater the risk of insomnia among adolescents (OR 5, 95% CI 1.21-1.99), and there is a clear dose-response relationship [47]. Second, UPF consumption may lead to disturbances in the intestinal flora and increase stress susceptibility of adolescents through the hypothalamic-pituitary-adrenal axis, which can lead to depressive symptoms occurring [5]. Experimental studies have shown that excessive intake of UPF leads to a reduced abundance of potentially beneficial butyrogenic microbes (*Roseburia*, *Faecalbacterium prausnitzii*, *Lactobacillus*, *Bifidobacterium*, and *Lachnospiraceae*), and the abundance of potentially pathogenic or opportunistic microorganisms (*Alistiopsis and Pasteurella*) increases [48]. Finally, the intake of UPF leads to an increase in chronic inflammatory levels, which causes the occurrence of depressive symptoms [49]. Adolescents with UPF energy consumption ≥30% (tertile 3 of UPF) had a 79% increase in interleukin-8 levels and had higher C-reactive protein levels when compared with adolescents in tertile 1 of UPF [50]. UPF intake may lead to a dysregulation of immune cell homeostasis and inflammation, including decreased levels of anti-inflammatory cytokines and increased levels of proinflammatory cytokines and chemokines [51].

Adolescents have great potential for physical, cognitive, and social development, and adolescence is regarded as the second critical window after infancy [52]. The mental health and growth of adolescents are related to the future of a country and require extensive attention. Adolescent depression is a global concern. Adolescence is a period of rapid emotional and cognitive development and critical life transitions. Notably, the treatment of depression is expensive. A Netherlands study of 11- to 18-year-olds with subclinical depressive symptoms showed that the social costs associated with depressive symptoms amounted to €54 million (US $57 million) per year [53]. Therefore, prevention and early intervention of adolescent depression is a top priority, using strategies that target predisposing factors, causes, and depression symptoms. Modifiable risk factors are critical for reducing depression prevalence among adolescents. Eating behavior interventions have been recommended as an effective measure to prevent mental illness [31]. Therefore, it may be necessary to expand the monitoring of UPF consumption behaviors and depressive symptoms in adolescents, strengthen health education in schools and families, and promote healthy eating environments in schools to reduce UPF consumption and improve depressive symptoms in adolescents.

### Strengths and Limitations

This study has several strengths. First, the sample comprised adolescents from 11 counties in Yunnan Province, China, with good representativeness and extrapolation of research results. Second, this study was the first to report the association between UPF consumption and depressive symptoms in Chinese multiethnic adolescents. Finally, we used a variety of models to analyze the association between UPF consumption and depressive symptoms and obtained stable results.

This study also has a few limitations. First, information bias was inevitable in the investigation, such as recall and social expectation biases. Second, the current assessment of UPF intake relies on frequency data without quantification in terms of grams or caloric contribution, which is limited for an accurate analysis of the association between the precise intake of UPF and depressive symptoms in adolescents. Finally, cross-sectional data cannot determine causal associations, and bidirectional associations may need to be clarified in further cohort studies.

### Conclusion

This study found that the consumption of instant foods, carbonated beverages, and energy beverages was related to depressive symptoms among Chinese multiethnic adolescents. It is important to note that the observed association may have a reverse correlation, where depressive symptoms could lead to an increase in the intake of ultrapalmitic fatty acids among adolescents. This needs to be further confirmed in cohort studies. The results highlighted the importance of reducing UPF consumption among adolescents. These findings should be replicated in cohort studies to determine the causal relationship and the direction. The government, schools, and families should take effective measures to reduce UPF consumption among Chinese multiethnic adolescents to support mental health development in adolescence.

## Appendix

Multimedia Appendix 1 Results of the generalized linear model analysis of the association between ultraprocessed food consumption and depressive symptoms in adolescents without adjusting for variables.

## Abbreviations

CDI: Children’s Depression Inventory
GLM: generalized linear model
OR: odds ratio
RCS: restricted cubic spline
SSB: sugar-sweetened beverages
UPF: ultraprocessed food

## Data Availability

Data are available upon reasonable request.
