# Supplementary material for: Ultraprocessed Foods Associated With Depressive Symptoms Among Chinese Adolescents: Cross-Sectional Study
Source: JMIR Public Health Surveill. 2025 Oct 20;11:e75061. doi: 10.2196/75061 (PMC12583942; doi:10.2196/75061)
Supplement: Multimedia Appendix 1 [file publichealth_v11i1e75061_app1.docx]

**Table S1.** Association of UPF consumption with depressive symptoms in adolescents.

| Adolescents | Variable | *β* | SE | 95%*CI* | Wald *χ^2^* | *P* |
| --- | --- | --- | --- | --- | --- | --- |
| All participants | Staple processed food | -0.12 | 0.05 | -0.22－-0.02 | 5.99 | 0.014 |
|  | Processed meat | -0.11 | 0.05 | -0.20－-0.02 | 5.97 | 0.015 |
|  | Processed eggs | -0.03 | 0.05 | -0.13－0.08 | 0.23 | 0.632 |
|  | Preserved fruits | -0.03 | 0.06 | -0.14－0.08 | 0.30 | 0.582 |
|  | Instant foods | 0.33 | 0.05 | 0.23－0.43 | 40.31 | <0.01 |
|  | Salted snacks | 0.05 | 0.04 | -0.04－0.14 | 1.27 | 0.259 |
|  | Sweet snacks | 0.01 | 0.05 | -0.08－0.10 | 0.04 | 0.837 |
|  | Carbonated beverages | 0.21 | 0.05 | 0.12－0.31 | 20.64 | <0.01 |
|  | Juice beverages | -0.14 | 0.05 | -0.25－-0.04 | 7.56 | 0.006 |
|  | Energy beverages | 0.16 | 0.06 | 0.04－0.28 | 6.99 | 0.008 |
|  | Milk beverages | -0.05 | 0.05 | -0.15－0.04 | 1.20 | 0.272 |
|  | Other SSB | 0.28 | 0.06 | 0.17－0.39 | 23.39 | <0.01 |
| Boys | Staple processed food | -0.17 | 0.06 | -0.30－-0.05 | 7.49 | 0.006 |
|  | Processed meat | -0.06 | 0.06 | -0.17－0.06 | 0.96 | 0.328 |
|  | Processed eggs | 0.08 | 0.07 | -0.06－0.21 | 1.27 | 0.261 |
|  | Preserved fruits | 0.01 | 0.07 | -0.14－0.16 | 0.02 | 0.896 |
|  | Instant foods | 0.23 | 0.07 | 0.10－0.36 | 11.56 | 0.001 |
|  | Salted snacks | 0.03 | 0.06 | -0.09－0.15 | 0.24 | 0.622 |
|  | Sweet snacks | 0.04 | 0.06 | -0.08－0.16 | 0.39 | 0.531 |
|  | Carbonated beverages | 0.23 | 0.06 | 0.11－0.35 | 14.73 | <0.01 |
|  | Juice beverages | -0.14 | 0.07 | -0.27－-0.01 | 4.21 | 0.040 |
|  | Energy beverages | 0.23 | 0.08 | 0.08－0.38 | 8.89 | 0.003 |
|  | Milk beverages | -0.21 | 0.07 | -0.34－-0.07 | 9.17 | 0.002 |
|  | Other SSB | 0.27 | 0.08 | 0.13－0.42 | 13.27 | <0.01 |
| Girls | Staple processed food | -0.04 | 0.07 | -0.18－0.11 | 0.27 | 0.604 |
|  | Processed meat | -0.12 | 0.07 | -0.26－0.02 | 2.71 | 0.100 |
|  | Processed eggs | -0.14 | 0.08 | -0.30－0.02 | 2.78 | 0.095 |
|  | Preserved fruits | -0.15 | 0.08 | -0.31－0.02 | 3.15 | 0.076 |
|  | Instant foods | 0.48 | 0.08 | 0.33－0.64 | 36.62 | <0.01 |
|  | Salted snacks | 0.01 | 0.06 | -0.12－0.13 | 0.02 | 0.901 |
|  | Sweet snacks | -0.07 | 0.06 | -0.19－0.06 | 1.10 | 0.294 |
|  | Carbonated beverages | 0.37 | 0.08 | 0.22－0.52 | 23.26 | <0.01 |
|  | Juice beverages | -0.20 | 0.08 | -0.35－-0.04 | 6.20 | 0.013 |
|  | Energy beverages | 0.31 | 0.10 | 0.11－0.51 | 9.42 | 0.002 |
|  | Milk beverages | -0.03 | 0.07 | -0.16－0.11 | 0.13 | 0.722 |
|  | Other SSB | 0.28 | 0.09 | 0.11－0.45 | 9.94 | 0.002 |

Unadjusted for variables.
